# Integrative linkage mapping and transcriptomic profiling uncover ozone-response modules in a peri-urban forest tree

**DOI:** 10.1093/g3journal/jkag069

**Published:** 2026-03-25

**Authors:** Xochitl Granados-Aguilar, Verónica Reyes-Galindo, Gustavo I Giles-Pérez, Jaime Gasca-Pineda, Alicia Mastretta-Yanes, Juan Pablo Jaramillo-Correa

**Affiliations:** Departamento de Ecología Evolutiva, Instituto de Ecología, Universidad Nacional Autónoma de México, Mexico City, CDMX 04510, Mexico; Posgrado en Recursos Genéticos y Productividad–Genética, Colegio de Postgraduados, Campus Montecillo, Texcoco, State of Mexico 56264, Mexico; Departamento de Ecología Evolutiva, Instituto de Ecología, Universidad Nacional Autónoma de México, Mexico City, CDMX 04510, Mexico; Programa de Doctorado en Ciencias Biológicas, Universidad Nacional Autónoma de México, Mexico City, CDMX 04510, Mexico; Departamento de Ecología Evolutiva, Instituto de Ecología, Universidad Nacional Autónoma de México, Mexico City, CDMX 04510, Mexico; Departamento de Ecología Evolutiva, Instituto de Ecología, Universidad Nacional Autónoma de México, Mexico City, CDMX 04510, Mexico; Ecosystem Stewardship Department, Royal Botanic Gardens, Kew, Richmond, London TW9 3AB, United Kingdom; Departamento de Ecología Evolutiva, Instituto de Ecología, Universidad Nacional Autónoma de México, Mexico City, CDMX 04510, Mexico

**Keywords:** *Abies*, peri-urban forests, co-regulation, transcriptomics, stress response, ozone pollution, Pinaceae

## Abstract

The genus *Abies* Mill. (Pinaceae) comprises a group of conifers distributed across boreal and temperate regions, including eight species with disjunct distributions across Mexico's highest mountain chains. *Abies religiosa* (Kunth) Schltdl. & Cham. is a dominant species of the Trans-Mexican Volcanic Belt in central Mexico, forming forests crucial for water retention, carbon sequestration, and soil stabilization. Despite its ecological importance, peri-urban forests dominated by this species around Mexico City are exposed to high levels of tropospheric ozone, which cause premature senescence and forest decline. Here, we report a saturated linkage map for *A. religiosa* generated by genotyping 182 megagametophytes from two mother trees for 9,702 single nucleotide polymorphisms. The linkage map is composed of 12 linkage groups (LGs) containing between 518 and 1,207 markers and spanning 1,567.88 cM (114 to 208 cM per LG). Annotation of reads containing SNPs allowed us to locate 5,881 coding genes on the map, of which 1,952 had known functions in conifers. Differential expression analyses of these genes in symptomatic and asymptomatic trees growing in a peri-urban forest heavily affected by ozone pollution revealed eight genes that were differentially expressed when ozone levels increased. Co-expression analyses further showed that neighboring genes tended to co-express more often in symptomatic than in asymptomatic trees, especially in clusters within LGs 5, 8, and 10. Gene pairs within co-expression clusters coded for similar proteins, suggesting functional co-localization. Our integrated approach reveals previously uncharacterized metabolic and defense pathways associated with ozone tolerance in conifers and lays the groundwork for developing molecular-based management programs accounting for ozone resistance in peri-urban forests.

## Introduction

High-throughput sequencing techniques, such as genotyping-by-sequencing (GBS), have enabled the generation of large numbers of SNPs for nonmodel species. This has greatly facilitated phylogenetic, phylogeographic, ecological, and functional genomic studies and the construction of highly saturated linkage maps (eg Parchman et al. 2018; Rudman et al. 2018; Feng et al. 2024). Such approaches are particularly valuable for species with extremely large and repetitive genomes that pose significant challenges for genome assembly, like conifers, whose genomes are often in excess of 30 Gb (Gagalova et al. 2022; Rodríguez et al. 2022). In this group of plants, linkage mapping is further facilitated by the use of megagametophytes, the haploid seed tissue that surrounds the embryo. This tissue originates from a single meiotic event, and it is strictly maternally inherited, which allows the identification of recombination and segregation events by using seeds from open-pollinated trees, thereby eliminating the need for controlled crosses and the production of an F2 generation (Tulsieram et al. 1992).

Although chromosome-level genome assemblies are already available for a few conifers (eg Niu et al. 2022; Lou et al. 2023), many reference genomes for this group of plants are still in draft form. Completely sequencing and correctly assembling such large and repetitive genomes require significant funding and extensive bioinformatic resources, which are not available to most research groups, especially in the Global South, where many endemic conifers are distributed. The use of linkage maps is thus still a necessary step for guiding conifer genome assembly (Tumas et al. 2024). Once constructed, linkage maps facilitate marker-trait association analyses and help identify genomic regions associated with phenotypic variation (Kulwal 2018). In conifers, map-based association studies have mainly focused on traits important for tree breeding, like fast growth, high wood quality, or pest resistance (Ritland et al. 2011; Lebedev et al. 2020). However, in response to the current environmental crisis, a necessary shift toward identifying regions linked to drought resistance and pollution tolerance is required (Moran et al. 2017; Baldi and La Porta 2022). Such studies have been carried out on crops (eg Wang et al. 2017; Geng et al. 2023; Wu et al. 2023), but they are still scarce in forest trees (but see Jia et al. 2020; De La Torre et al. 2022).

Linkage maps can also be integrated with transcriptomic data, allowing the mapping of gene expression and the identification of co-expressed genes involved in cascade responses to stress (van Dam et al. 2017; Wang et al. 2017; Geng et al. 2023). This integration is still scarce in forest trees (eg Pavy et al. 2017). Combining these approaches could be particularly beneficial for species affected by sudden environmental threats, such as drought or soil and air pollution, as it could provide initial insights into the genomic location of co-expressed genes involved in responses and potential adaptation to such abrupt environmental pressures.

Firs belong to the genus *Abies* Mill., one of the most diverse groups within the Pinaceae family, with approximately 48 species (Farjon 1990). They are part of the Abietoideae subfamily (together with cedars and hemlocks), which diverged approximately 200 Ma from the Pinoideae subfamily (Stull et al. 2021). This last subfamily contains most of the conifer species studied from an evolutionary genomics perspective to date (spruces, pines, and larches; Nystedt et al. 2013; De La Torre et al. 2014; Niu et al. 2022), which highlights the need for developing novel genomic resources with broader taxonomic coverage.

Distinguished by their esthetic appeal and significant economic, cultural, and ecological roles, firs are primarily distributed across boreal and temperate regions, with some species extending into tropical/subtropical mountain ecosystems (Farjon 1990; Xiang et al. 2009). A major center of fir diversity is located between the southwestern United States and Guatemala, where approximately 14 species occur, including 8 taxa with disjunct distributions in the highest Mexican mountain ranges (Rzedowski 2006; Aguirre-Planter et al. 2012; Semerikova and Semerikov 2014).

In Mexico, fir forests provide crucial ecosystem services, such as water capture and filtration, oxygen production, carbon sequestration, and soil retention (Martínez-Méndez et al. 2016). Among these species, sacred fir, *Abies religiosa* (Kunth) Schltdl. & Cham., stands out as one of the most prevalent and iconic species in the central parts of the country. It is distributed along an east-west ecological gradient in the Trans-Mexican Volcanic Belt, at elevations between 2,500 and 3,100 m asl (Rzedowski 2006). Moreover, forests dominated by this species are the preferred overwintering habitat of the monarch butterfly (*Danaus plexippus* L.) and hold cultural significance in religious ceremonies (Farjon 2010).

Sacred fir forests are particularly threatened by illegal logging, wildfires, climate change, water stress, and environmental pollution (Shao et al. 2017; Vivar-Vivar et al. 2021; Sáenz-Romero et al. 2024). A large part of this pollution takes the form of tropospheric ozone (O_3_), which is produced by a chemical reaction between byproducts of fossil fuel burning triggered by ultraviolet light (Riveros et al. 1998; Baumgardner et al. 2012; Peralta et al. 2021). In firs, ozone exposure results in visible foliar symptoms that include chlorophyll degradation and chlorotic mottling, which leads to the development of large reddish lesions and premature needle senescence (Peterson et al. 1987; Patterson and Rundel 1993; Alvarez et al. 1998; Reyes-Galindo et al. 2024). Due to the continuous incidence of ozone, 35% of fir trees from Mexico City's peri-urban forests show symptoms of ozone damage (Reyes-Galindo et al. 2023).

In a previous study, we documented one of the first cases of ozone tolerance in conifers by investigating the histological, metabolomic, and transcriptomic responses of asymptomatic (healthy) and symptomatic (damaged) sacred firs during periods of moderate (87 ppb) and high (170 ppb) concentrations of tropospheric ozone (Reyes-Galindo et al. 2024). Here, we combine this available RNA-sequence data with a newly generated and annotated linkage map for *A. religiosa*. We were aiming to identify gene clusters involved in co-expression patterns associated with ozone tolerance by comparing data from healthy and ozone-damaged trees. In addition to identifying these regions, we lay the groundwork for future genome wide association studies and an eventual genome assembly for sacred fir; together, these efforts will support the development of informed reforestation strategies in polluted peri-urban environments.

## Methods

### Sampling, DNA extraction, and sequencing

Cones were collected from 2 mature and dominant *A. religiosa* trees in 2 natural stands, one located at San Rafael Ixtapalucan, Puebla (19°15′31.0″N 98°36′37.0″W), where no ozone exposure is expected, and another one at Santa Rosa Xochiac, Mexico City (19°18′20.9″N 99°18′40.8″W), within a peri-urban forest affected by ozone pollution. These localities are separated by 112 km, on different mountain peaks. Cones were dried in paper bags with silica gel, and seeds were collected after cone disintegration. Seeds were cleaned with 70% alcohol and soaked in double-distilled water overnight to facilitate dissection. The haploid megagametophyte was separated from the seed coat and the embryo for 95 seeds per “mother tree” and stored at −70 °C. DNA was extracted from each megagametophyte with the Qiagen DNeasy Plant kit. DNA quality was evaluated by agarose gel electrophoresis, and its quantity was determined using a Qubit 4 fluorometer (Thermo Scientific).

Genomic DNA (20 ng/µL per sample) was double-digested with restriction enzymes *Pst*I and *Msp*I, and unique barcodes were assigned to each sample. GBS libraries were prepared, after size selection with a Blue Pippin Prep machine (SAGE Sciences), and sequenced (paired-end) on an Illumina NovaSeq 600 system at the “Plateforme d'analyses génomiques, Institut de Biologie Intégrative et des Systèmes” (IBIS, Université Laval, Québec, Canada), following the standard protocols for plants with large and complex genomes (Poland et al. 2012; Boudhrioua et al. 2017).

### SNP calling, linkage map construction, and marker annotation

Sequence reads were demultiplexed (GBSX v1.3; Herten et al. 2015) and quality-checked (MultiQC v1.32; Ewels et al. 2016). After adapter and quality trimming (Trimmomatic v0.39; Bolger et al. 2014) and length standardization to 120 bp (Cutadapt v5.2; Martin 2011), loci were assembled *de novo* using Stacks2 (v2.68; Rochette et al. 2019) using the denovo_map.pl pipeline and following previous GBS workflows in *A. religiosa* (Giles-Pérez et al. 2022; Arenas et al. 2025). SNPs were then called and genotyped from the assembled loci, and VCF files were exported with the Stacks populations module (including –vcf and –vcf-all options). We excluded samples with fewer than 500,000 reads (scripts available at https://github.com/Xochitl-Citlalmina/Abies-linkage-map-transcriptome).

Filtering and linkage map construction were performed in Lep-MAP3 v0.2 (Rastas 2017) using the recommended module order (ParentCall2, Filtering2, SeparateChromosomes2, JoinSingles2All, and OrderMarkers2). Given the haploid nature of the data, markers were filtered using a heterozygote rate of 0.05 and a segregation distortion threshold at 0.001. LGs were defined with lodLimit = 30 and distortionLod = 1, recovering 12 LGs consistent with the haploid chromosome number of *Abies* (Rice et al. 2015). Marker ordering was carried out with selfingPhase = 1, and the final maps were selected based on likelihood and standard ordering diagnostics (Rastas 2017) (further details and scripts are available at the GitHub repository cited above).

Marker order was evaluated using the squared logarithm of odds (LOD) score matrix computed in Lep-MAP2 v0.2 (Rastas et al. 2015) and visualized as heatmaps (gnuplot; Phillips 2012), following established recommendations for well-ordered linkage maps. Final LGs were selected based on combined criteria of maximum likelihood and consistent LOD heatmap patterns (signal concentrated along the diagonal), and the final map representation was prepared in Genetic-Mapper (Bekaert 2016). A goodness-of-fit assessment of within-LG marker counts in 10- and 20-cM bins against Poisson and negative binomial expectations was performed in R (R Core Team 2025); full details are provided in the GitHub repository above.

FASTA sequences were exported from Stacks and annotated by BLAST searches (word size = 11; *E*-value < 1e-30; Camacho et al. 2009) against the transcriptomes of *Abies balsamea* (L.) Mill. (Van Ghelder et al. 2019) and *A. religiosa* (Reyes-Galindo et al. 2024). Significant hits were functionally characterized using InterProScan v5.74 (Jones et al. 2014) and The Arabidopsis Information Resource (TAIR; Berardini et al. 2015). All commands and custom scripts are available on GitHub and Zenodo (DOI: 10.5281/zenodo.17923287).

### Testing for the co-expression of neighboring genes

We tested for correlated expression between closely located genes in the linkage map. To do so, we used the RNA-seq data from *A. religiosa* trees located in a peri-urban forest west of Mexico City that is constantly exposed to harmful tropospheric ozone concentrations, which had previously been aligned to our linkage map using blast. These data were obtained from the young needles of 10 trees, aged between 10 and 15 years, that were sampled during periods of moderate (87 ppb; 15 April 2017) and elevated tropospheric ozone (170 ppb; 17 May 2017). Five trees showed symptoms of ozone-related stress, and the other 5 were asymptomatic (Reyes-Galindo et al. 2024).

RNA library preparation and sequencing were performed on the same platform as the GBS analyses described above. Reads were demultiplexed, filtered, mapped to the *A. balsamea* transcriptome, and annotated with TRAPID v2.0 (see Reyes-Galindo et al. 2024, and Supplementary Table 1 for all details). Our workflow of analyses consisted of first matching the mapped genes of the *A. religiosa* linkage map to this RNA-seq data and then examined co-expression patterns between neighboring genes on the linkage map. Finally, we performed differential expression analyses between adjacent genes.

Neighboring genes are expected to form groups with similar expression patterns (Boutanaev et al. 2002; Freeling 2009; Nazari and Zinati 2023). We aimed to test whether such groups could be identified when tropospheric ozone exposure increased and whether they differed between symptomatic and asymptomatic trees. To identify regions of co-expressed genes, we first obtained, from the RNA-seq data, the number of reads per gene using a bash script (https://doi.org/10.5281/zenodo.17923287). Subsequently, in R, we extracted the gene names, expression counts, and linkage group positions. Using Python v3.11 (Python Software Foundation 2024 Sept 7), we calculated the Pearson correlation between genes at intervals of 1 cM per linkage group and mapped such values onto the linkage map. To reduce background noise for weighted gene co-expression network analysis (WGCNA) (Langfelder and Horvath 2008), we preprocessed the gene expression data by retaining only those genes with expression levels of 10 or higher in at least 50% of the samples. The data were then transformed for co-expression network analysis in WGCNA, with samples as rows and genes as columns. Outliers and missing values were removed. We chose a soft-thresholding power that was close to scale-free topology (*R*^2^ ≥ 0.8). Then, we used hierarchical clustering and dynamic tree cut to determine gene modules. We assigned each gene to a module and mapped it to its genetic position. This made it possible to measure how modules are spread out across LGs. Using the dplyr library (Wickham et al. 2023) in R v4.4.1, we calculated Pearson correlation coefficients between all pairs of co-expressed genes in each module and LG, based on a given genetic distance (ie ≤1 cM and ≤5 cM), and adjusted for false discovery rate (FDR < 0.05) to find significant correlations. Significant gene pairs were plotted using igraph (Antonov et al. 2023) (Supplementary Fig. 5). Lastly, a *χ*² test was performed on the module-by-linkage group contingency table to establish how the modules were distributed on the linkage map (Supplementary Table 5). We anticipated that the number of co-expressed genes would increase with ozone exposure and that asymptomatic trees would show more groups of co-expressed genes than symptomatic trees (ie those potentially involved in response to ozone stress). Differential expression analyses were performed in edgeR (Chen et al. 2025) and DESeq2 (Love et al. 2014) using R v4.4.1. We performed the following comparisons between samples: (i) symptomatic vs asymptomatic trees during the moderate ozone concentration period; (ii) symptomatic vs asymptomatic trees during the high ozone concentration period; and (iii) and (iv) trees with the same phenotype (symptomatic or asymptomatic) during the moderate vs the elevated ozone concentration periods.

## Results

### SNP calling and linkage map

We generated 485,508,095 reads for 190 *A. religiosa* megagametophytes. Eight samples from the Puebla mother tree were eliminated because of low read numbers (<500 K reads). Read numbers for the 182 retained samples ranged from 516,368 to 14,501,887 (average 2,667,771 reads); they had an average quality score of 36 and a mean guanine-cytosine content of 55%. After alignment, clustering, and correction, 256.93 million high-quality reads were retained and assigned to 1.26 million loci. The average depth coverage per locus was 13.9 × for the Puebla megagametophytes and 36.7 × for the Mexico City samples.

**Fig. 1.**
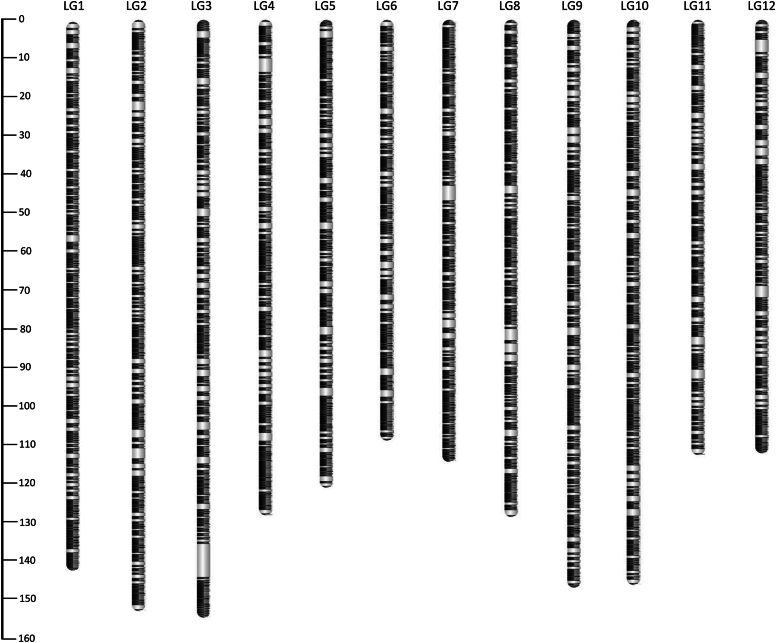
Ideogram representing the 12 linkage groups of the composite map of *A. religiosa*; mapped markers (*n* = 9,702) are denoted as black lines, and the scale on the left is in centimorgans (cM).

**Fig. 2.**
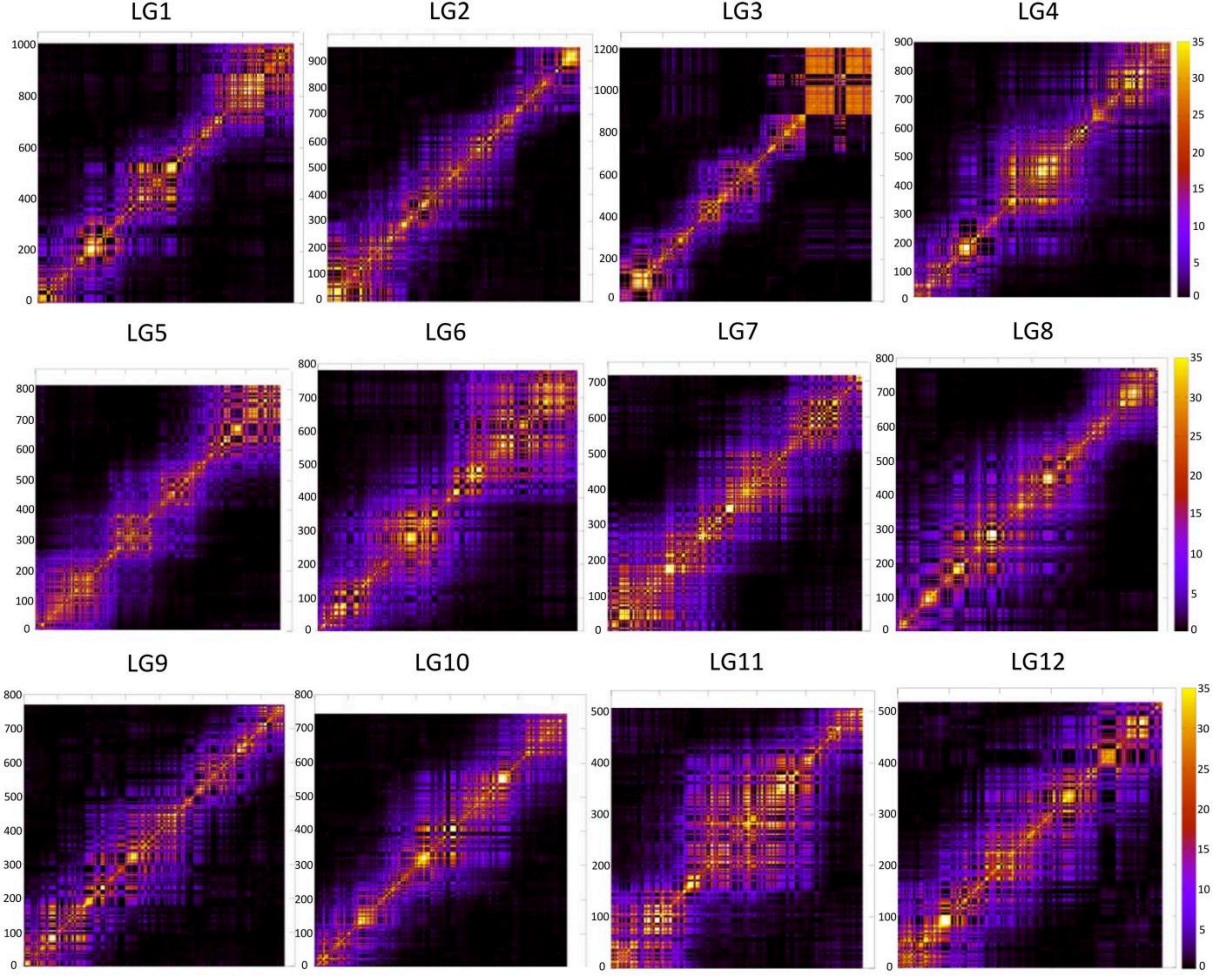
Plot for the LOD score square matrix of each linkage group of the composite linkage map of *A. religiosa*; lighter tones in the middle of each square indicate good marker distribution.

After de novo calling, 536,365 SNPs were obtained, of which 187,378 were informative. After filtering for segregation distortion (*P* = 0.001), 10,262 SNPs were retained for map construction, of which 9,702 could be assigned and organized into 12 LGs; this number matches the haploid chromosome number of *Abies* (Rice et al. 2015). The total map length was 1,567.88 cM (Fig. 1), and the number of markers within LGs ranged from 518 to 1,207. LGs had lengths ranging from 108.74 to 154.68 cM (Table 1), and markers within LGs had an average distance of 0.16 cM. The dot plots obtained from Lep-MAP3 helped order markers and identify mapping errors (ie when red lines were observed). Such errors were observed for LGs 7, 8, and 11, for which we selected the map with the second-best likelihood, which did show no red lines in the dot plot. The final dot plots are shown in Supplementary Fig. 1. Across LGs, the LOD score matrices showed a pattern of concentrated signal along the diagonal, indicating a consistent marker order and supporting the final linkage group arrangement (Fig. 2). Markers exhibited a structured distribution within LGs that did not follow a Poisson distribution when surveying bins of both 10 and 20 cM (Supplementary Fig. 2).

**Table 1.** Description of the *A. religiosa* composite map (Kosambi distances), including map length, SNP count, average inter-marker distance, and the number of unique map positions (cM) (the number of distinct cM coordinates per linkage group), co-located SNPs (SNPs sharing the same cM coordinate), and annotated genes per linkage group.

LG	Length (cM)	Nb of SNPs	Average distance between SNPs (cM)	Nb of unique map positions (cM)	Nb of SNPs mapped to the same cM position	Nb of annotated genes per LG
LG1	141.96	1004	0.1415	257	912	208
LG2	152.97	953	0.1606	254	867	189
LG3	154.68	1207	0.1282	236	1126	181
LG4	128.11	899	0.1426	224	808	168
LG5	120.97	815	0.1486	210	744	154
LG6	108.74	782	0.1392	193	713	155
LG7	114.28	718	0.1593	209	627	162
LG8	128.56	772	0.1667	229	681	192
LG9	146.88	771	0.1907	244	676	148
LG10	146.18	745	0.1964	257	638	161
LG11	112.46	518	0.2175	183	440	120
LG12	112.03	518	0.2167	182	448	114
Total	1567.88	9702	0.1618	2,678	8,680	1,952

### Gene annotation and co-expression under air pollution stress

Following BLAST searches, we were able to annotate 6,036 SNPs out of 9,702 and assigned them to 5,881 coding genes (Supplementary Table 2). Using InterPro and TAIR, we were able to assign known protein functions to 1,952 of such genes; this is summarized in Table 1 and can be consulted in detail in Supplementary Table 3. The average number of annotated genes per linkage group was 162.67, with a range of 114 to 208 genes per LG; the average distance between neighboring genes within LGs was 0.7535 cM. We identified 64 repeated genes within the same LG, most of which had 2 copies. Exceptions included the *TPR-like*, the *BGLU44 B-S* glucosidase, the *H2B* histone, and the *ARM* repeat proteins, which had 3 copies each, as well as the *PME2* pectin methylesterase family, which was repeated 5 times (LG3). We also observed 77 cases of gene copies located in multiple LGs, most of which included large plant gene families like the *LRR* receptor kinases*, TPR proteins,* and *UDP-*glycosyltransferases. We observed no pattern in the distribution of these genes across the 12 LGs (Supplementary Tables 3 and 4).

**Fig. 3.**
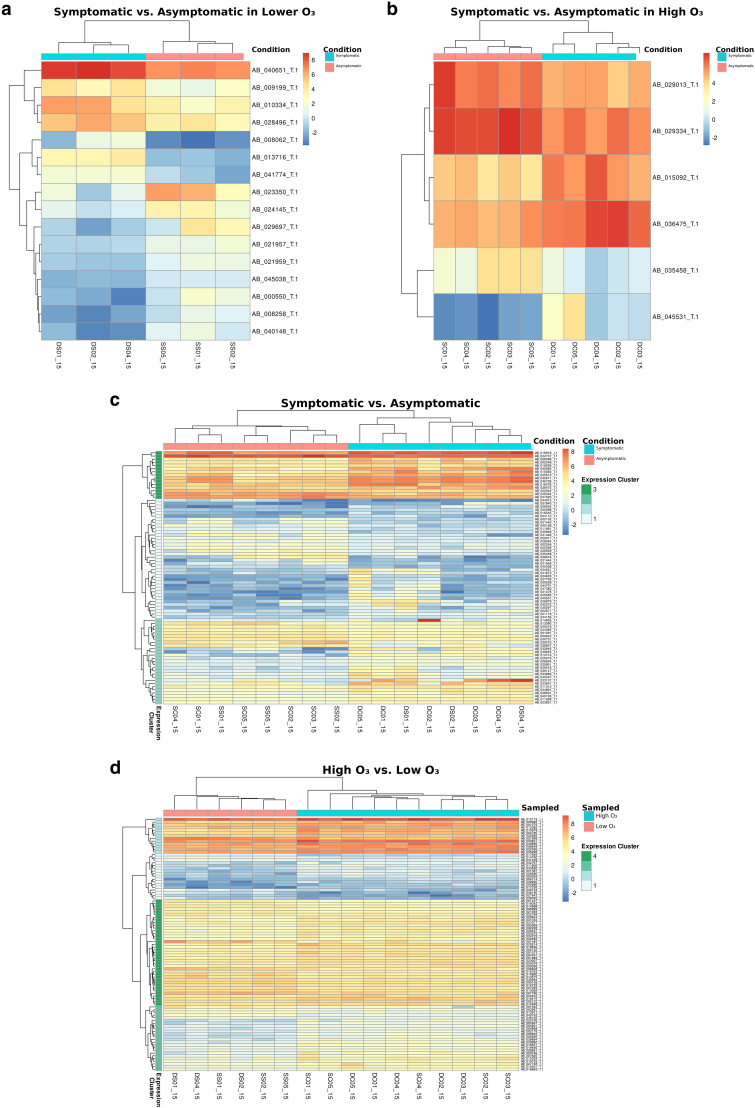
Gene expression profiles of symptomatic (damaged) and asymptomatic (healthy) *A. religiosa* natural trees during 2 ozone (O_3_) concentration periods, as determined with edgeR (see Supplementary Fig. 3 for results obtained with DESeq2). a) Differential gene expression between symptomatic and asymptomatic trees during the low-ozone period. b) Differential gene expression between symptomatic and asymptomatic trees during the high ozone period. c) Overall comparison of gene expression between symptomatic and asymptomatic trees, regardless of ozone concentration. d) Comparison of gene expression profiles during the high and low-ozone concentration periods, irrespective of the tree phenotype. Color scales represent normalized expression levels, with clustering indicating patterns of similarity across samples and genes.

After removing 1 sample (SS01_15), which recurrently showed deviant expression, we observed that most differences in gene expression occurred between symptomatic (damaged) and asymptomatic (healthy) samples, with ozone concentration emerging as a secondary factor influencing expression profiles (Fig. 3). During the moderate ozone concentration period (Fig. 3a), the most differentially expressed gene between asymptomatic and symptomatic trees was AB_023350, a *CXE18 carboxylesterase* located on LG5, which was upregulated in asymptomatic plants and downregulated in symptomatic ones. Other differentially expressed genes included AB_010334, AB_013716, and AB_028496, all of which were mostly upregulated in symptomatic trees; none of these 3 genes had a functional annotation in InterPro or TAIR. During the high ozone concentration period (Fig. 3b), only 1 gene showed a differential expression between the asymptomatic and the symptomatic trees (AB_035468T.1, no functional annotation), while 4 genes (AB_029013T.1, a hypothetical protein; AB_029334T.1, an *L-type lectin receptor kinase*; AB_015092T.1, a *nuclear fusion defective 4*; and AB_036475T.1, a *chitinase*) were upregulated in both plant groups (Fig. 3b). This suggests more a generalized rather than a differential response between the symptomatic and asymptomatic trees when tropospheric ozone concentrations increase.

To further refine our analysis, we re-examined the RNA-seq data by restricting the dataset to genes with known positions anchored to our high-density linkage map, rather than considering the full transcriptome as in Reyes-Galindo et al. (2024). Using this improved approach, we implemented a contrast design (including 4 phenotype–ozone comparisons) that allowed us to identify differentially expressed genes distributed along the LGs and to integrate these findings with co-expression modules and correlation clusters. By focusing on mapped genes, we were able to associate gene expression changes within specific genomic regions, thereby providing a more precise characterization of the molecular responses underlying ozone stress response. In the overall comparison between asymptomatic and symptomatic samples (ie independently of ozone concentration; Fig. 3c), asymptomatic plants had significantly fewer upregulated genes than the second group, which could indicate differential basal activation of stress or damage response pathways between groups. When comparisons were made between ozone concentration periods (ie without regard for plant symptoms; Fig. 3d), no significant differences were observed, suggesting that gene expression changes are more strongly associated with the presence/absence of symptoms than with increased ozone exposure. Expression analyses performed with DESeq2 corroborated these patterns (Supplementary Fig. 3).

**Fig. 4.**
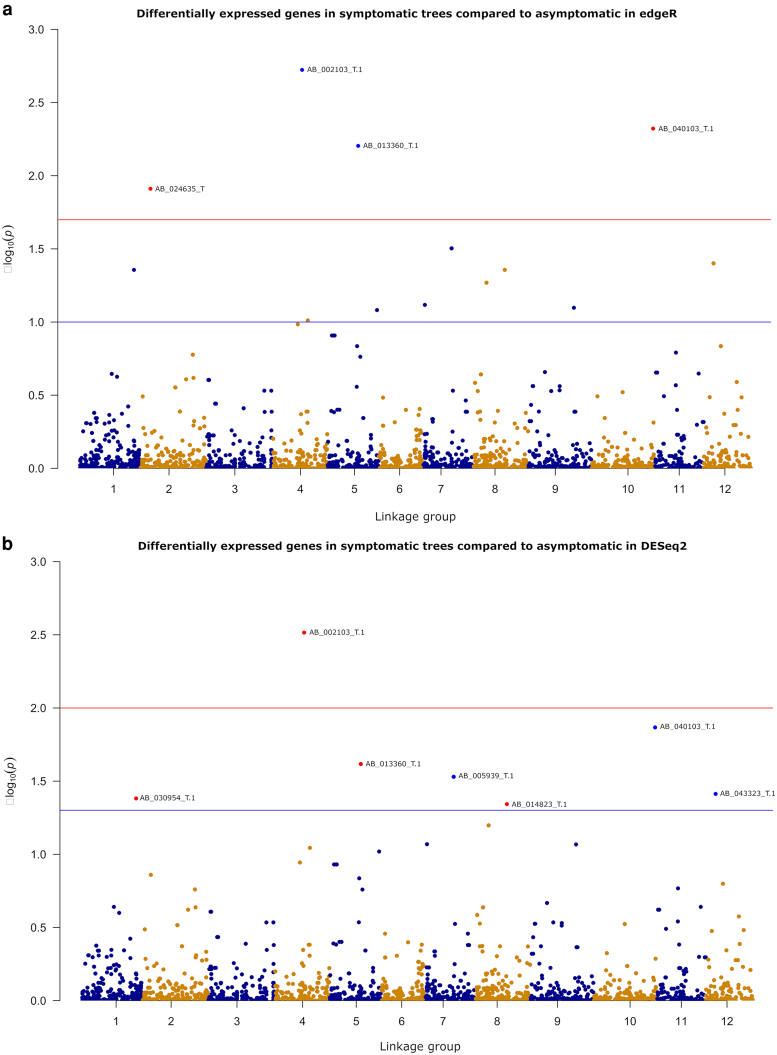
Manhattan plots showing the position of the annotated genes differentially expressed between the symptomatic and asymptomatic individuals on the composite linkage map of *A. religiosa*. Each point represents an annotated gene, with genes upregulated and downregulated in the symptomatic trees, respectively highlighted in red and blue. Labeled points indicate genes that exceed significance thresholds (horizontal lines). a) Genes identified using edgeR; b) genes identified using DESeq2.

Eight genes located on the linkage map showed significantly different expression levels between symptomatic and asymptomatic trees. Differential expression analysis identified these genes as significant, with edgeR detecting 4 genes and DESeq2 identifying 7. Each methodology revealed a partially overlapping but distinct set of differentially expressed genes. Three of these genes, AB_002103_T.1, AB_013360_T.1, and AB_040103_T.1, were detected by both methods. The location of these genes on the linkage map is shown in Fig. 4. They are distributed across various LGs without forming clear clusters: AB_030954_T.1 (*SAUR*-like auxin-responsive protein) on LG1, AB_024635_T.1 (no annotation) on LG2, AB_002103_T.1 (intrinsically disordered protein) on LG4, AB_013360_T.1 (*carboxylesterase 18*) on LG5, AB_005939_T.1 (*Rossmann-fold NAD(P)*-binding protein with disorder features) on LG7, AB_014823_T.1 (intrinsically disordered protein) on LG8, AB_040103_T.1 (*2-oxoglutarate and Fe (II)-dependent* oxygenase) on LG10, and AB_043323_T.1 (*chitinase*) on LG12. In terms of function, these genes can be divided into 2 main groups (see Table 2 for all functional annotations): (i) proteins involved in hydrolytic, oxidative, or defense-related activities; and (ii) intrinsically disordered proteins.

**Table 2.** Description of annotated genes that showed significantly different expression between symptomatic and asymptomatic *A. religiosa* trees, and significant co-expression in trees with a same phenotype. Genes marked with an asterisk (*) indicate significant differential expression in both edgeR and DESeq2, significantly co-expressed genes (identified through WGCNA) are shown in bold.

LG	Position average in cM	Gen	InterPro description	TAIR description
1	127.053	AB_030954_T.1*	No hit	SAUR-like auxin-responsive protein family
2	20.641	AB_024635_T.1*	No hit	No hit
4	67.729	AB_002103_T.1*	consensus disorder prediction	No hit
5	75.5055	AB_013360_T.1*	Alpha/Beta hydrolase fold	*AtCXE18*, *CXE18* carboxyesterase 18
7	64.082	AB_005939_T.1*	consensus disorder prediction	NAD(P)-binding Rossmann-fold superfamily protein
8	72.688	AB_014823_T.1*	consensus disorder prediction	No hit
10	139.6905	AB_040103_T.1*	consensus disorder prediction	2-oxoglutarate (2OG) and Fe (II)-dependent oxygenase superfamily protein
12	26.1855	AB_043323_T.1*	No hit	*ATEP3*, *ATCHITIV*, *CHIV, EP3* homolog of carrot *EP3-3* chitinase
5	79.1605	**AB_023350_T.1**	Alpha/Beta hydrolase fold	*AtCXE18*, *CXE18* carboxyesterase 18
8	27.602	**AB_002192_T.1**	No hit	Copper amine oxidase family protein
8	27.602	**AB_007527_T.1**	No hit	No hit
10	141.3755	**AB_005854_T.1**	Adenosylhomocysteinase-like superfamily	No hit
10	140.8135	**AB_024743_T.1**	Adenosylhomocysteinase-like	*SAHH2*, *ATSAHH2 S*-adenosyl-l-homocysteine (SAH) hydrolase 2

**Fig. 5.**
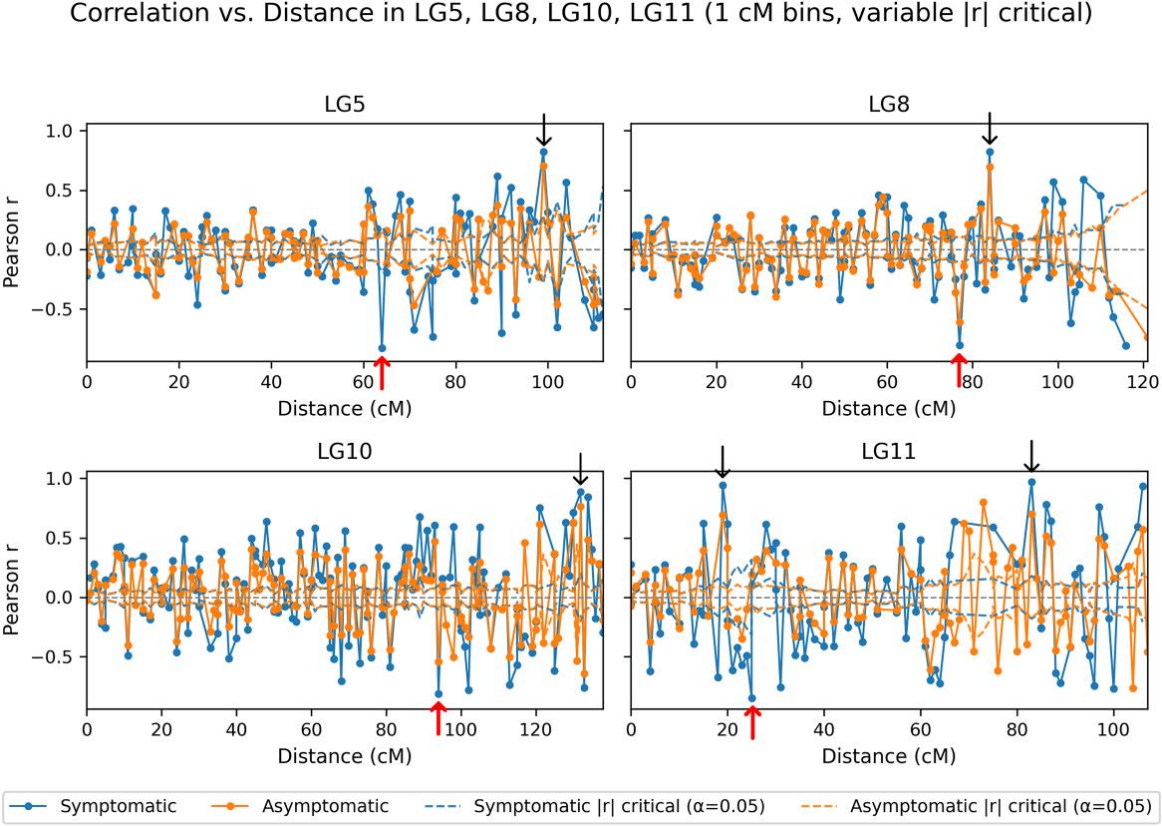
Pearson correlation coefficients (*r*) between the expression profiles of neighboring gene pairs (1 cM bins) along linkage groups (LG) 5, 8, 10, and 11 for symptomatic (blue) and asymptomatic (orange) *A. religiosa* trees (see Supplementary Fig. 4 for all 12 LGs). Dashed lines represent critical significance values at *α* = 0.05. Red arrows indicate regions with strong negative correlations, while black arrows indicate regions with strong positive correlations (see text for details).

Across all LGs, the correlation of expression values between neighboring genes separated by ≤1 cM often exceeded the |r| critical threshold, indicating gene co-expression (i.e. positive Pearson correlations) or antagonistic expression (ie negative correlations; Fig. 5 and Supplementary Fig. S4). Several peaks reaching |r| values well above/below 0.5 were observed along LGs 5, 8, 10, and 11 (black and red arrows on Fig. 5). The highest values occurred in LG11, whereas the lowest were observed in LGs 5 and 11. Interestingly, these correlated expression peaks were more pronounced and broader in symptomatic trees than in asymptomatic ones and included 2 peaks of antagonistic expression (in LG5 and LG11), with |r| values below −0.80, which were absent in asymptomatic individuals (Fig. 5).

When correlations were calculated for all gene pairs within the same LG, we identified sets of 59–85 strongly correlated genes per linkage group (Supplementary Table 4). Again, the transcripts involved in the highest number of correlations were located in LG5 (AB_012526_T.1, AB_007941_T.1, AB_022780_T.1), LG8 (AB_024572_T.1, AB_022464_T.1, AB_006073_T.1), LG10 (AB_030874_T.1, AB_046125_T.1, AB_003515_T.1), and LG11 (AB_012375_T.1, AB_015014_T.1, and AB_015934_T.1). This again suggests that co-expression modules coordinating the ozone stress response in sacred fir are likely located within these 4 LGs. Most of the genes above could not be annotated in InterPro or TAIR, except for AB_012526_T.1 (*FRA3*, an *endonuclease/exonuclease/phosphatase* protein), AB_007941_T.1 (a *protein kinase*), AB_022780_T.1 (a *pathogenesis-related thaumatin* protein), and AB_003515_T.1 (a *G2484-1* protein).

We then searched for co-expression networks using WGCNA, focusing our analyses on these 4 LGs. After correcting for FDR, we retained 3 pairs of genes with highly correlated expression profiles on LGs 5, 8, and 10 (Table 3). These gene pairs were less than 3.7 cM apart and included 2 *carboxyesterase* genes (AB_013360_T.1, AB_023350_T.1) on LG5; a *copper amine oxidase* (AB_002192_T.1) and a hypothetical protein (AB_007527_T.1) on LG8; and an *adenosylhomocysteinase*-like enzyme (AB_005854_T.1) and an unknown protein (AB_024743_T.1) on LG10.

**Table 3.** Pairs of significantly correlated genes identified in the WGCNA co-expression analysis after adjusting for false discovery rate.

LG	Gene1	Gene 2	Distance (cM)	Correlation	Bin	*P*-value	FDR
5	AB_013360_T.1	AB_023350_T.1	3.655	0.97628013	≤5 cM	1.35E-06	0.00481898
10	AB_005854_T.1	AB_024743_T.1	0.562	0.99153869	≤1 cM	2.22E-08	0.00015896
8	AB_002192_T.1	AB_007527_T.1	0	0.95765411	≤1 cM	1.34E-05	0.03190317

To synthesize our findings, we identified several differentially expressed genes between high- and low-ozone periods, located mainly (though not clustered) across LGs 5, 8, 10, and 11. Many of these genes—some unannotated and others with known defense or metabolic functions—showed strong co-expression or antagonistic patterns with neighboring genes, particularly in symptomatic trees. Network analyses further revealed 3 pairs of closely located genes forming the core of the local co-expression modules associated with response to ozone exposure (Supplementary Fig. 5). Overall, our results suggest that the ozone stress response in *A. religiosa* involves a coordinated transcriptional network distributed across multiple genome regions, comprising both known and potentially novel components involved in ozone adaptation.

## Discussion

Here, we are presenting the first linkage map for a Mexican fir (*A. religiosa)*, the second reported for the genus *Abies* (after that of *Abies sachalinensis*; Goto et al. 2017), and one of the first for a Mexican conifer from any genus (see also Shepherd et al. 2003; Yang et al. 2013). This highly saturated linkage map contains more than 9.7 K SNPs distributed across 12 LGs, matching the haploid chromosome number of firs (*n* = 12; Rice et al. 2015). Markers include 5.8 K SNPs located within coding genes, of which 1.9 K encode proteins with known functions. Such a number of mapped genes allowed us to combine LG map positions with transcriptome data and test co-expression hypotheses in trees exposed to tropospheric ozone in a peri-urban forest west of Mexico City. We identified 3 modules of co-expressed genes potentially involved in defense responses, including cellular detoxification and epigenetic regulation.

### Linkage map construction

Our high-density linkage map includes 9,702 markers distributed across 12 LGs, which significantly exceeds the 1,251 markers used to build the linkage map of *A. sachalinensis* (Goto et al. 2017), the only linkage map previously available for the genus *Abies*. Ultra-dense linkage maps have recently been developed for model conifers, such as *Picea abies* and *Picea sitchensis*, each comprising over 21,000 markers (Bernhardsson et al. 2019; Tumas et al. 2024); our map therefore exhibits an intermediate density, with a minimal average distance of 0.16 cM between adjacent markers. However, despite a lower total marker count than those for *Picea*, our linkage map contains a comparable number of coding regions to those mapped in *P. sitchensis* (5,414 distinct coding sequences), a species for which much more extensive genomic resources are available (Tumas et al. 2024). Such comparable gene coverage highlights the importance of selecting appropriate restriction enzymes for anonymous SNP generation and emphasizes the value of linkage maps based on such markers for genomics analyses in nonmodel conifers, especially when funding and genomic resources are limited.

The genetic markers used here were obtained using a methylation-sensitive enzyme that reduced the proportion of repetitive DNA and enriched our libraries for gene-coding regions (Pootakham et al. 2016; Pootakham 2023). Compared with exome capture, this approach does not require costly probe design or a priori transcriptome sequencing (Scheben et al. 2017), although we used an available annotated transcriptome from a related species (*A. balsamea*) as a reference for bioinformatic analyses (Van Ghelder et al. 2019). In addition, SNP discovery was not constrained by tissue-specific gene expression, as occurs in RNA resequencing-based linkage mapping (Pootakham et al. 2014). Furthermore, in contrast to earlier marker systems such as AFLPs and SSRs, which were low-throughput and often less transferable across species and even among populations within species (Mir et al. 2013), our approach generated high-resolution, codominant SNPs at a fraction of the cost and with greater scalability.

A remaining caveat for our study concerns marker portability for comparative applications. Across species, correspondence among maps is generally ensured during the early stages of marker development (ie SNP detection from transcriptome sequencing and integration into genotyping arrays; Pavy et al. 2013). While the mapped SNPs from this and previous studies in *Abies religiosa* could be used to develop such an array, a temporary solution is to infer marker correspondence through sequence similarity (eg BLAST against transcriptome resources). However, because uncertainty in orthology identification can limit fine-scale comparisons, we decided not to attempt cross-species map comparisons as usually reported for other conifers (eg Pelgas et al. 2006; de Miguel et al. 2015; Tumas et al. 2024). In the meantime, we provide the mapped locus sequences and annotations for *A. religiosa* (matches_loci.fa and lociLG_blast.gff3), which will facilitate treating these loci as sequence-defined anchors for future work with this and other Mexican firs.

The composite linkage map for sacred fir was constructed from 182 haploid megagametophytes, spans 1,567.88 cM, and has an estimated genetic genome coverage of ∼86.3%. These estimates were derived from the observed map length relative to the expected genetic genome length, following linkage-based estimators and using genome sizes reported for *Abies* (Chakravarti et al. 1991; Mosca et al. 2019). These values fall within the range observed in other conifers linkage maps, which exhibit considerable variation in map length and coverage. For instance, corresponding values were 976.5 cM and 55.25% for the *Taxodium* hybrid “Zhongshanshan” (Wang et al. 2016), 1,192.10 cM and 98.58% for *Pinus balfouriana* (Friedline et al. 2015), and ∼1,506 cM for a full-set RAD-seq map of *Platycladus orientalis* (with a shorter framework map after filtering; Jin et al. 2019). Such a variation among studies underscores the sensitivity of map length to marker selection thresholds (Jin et al. 2019). Importantly, genome coverage is not uniformly defined across studies, particularly when maps are designed for genome assembly, where coverage may reflect the fraction of scaffolds or gene models anchored rather than genetic coverage sensu stricto. Reported differences can also reflect population size, marker density, error filtering, and mapping algorithms. Within this heterogeneous landscape, our map provides a dense, gene-enriched framework suitable for downstream genomic and functional analyses in a nonmodel conifer.

Population size for linkage map construction also influences the number of detectable recombination events and, consequently, the resolution of tightly linked loci and the stability of local marker order. Although our 182 megagametophytes are sufficient to recover robust chromosome-scale LGs and support gene-level integration analyses, a larger sample would have allowed more precise fine-scale recombination estimates. For instance, genome assembly-oriented efforts in *Picea* used much larger linkage map populations (eg a diploid progeny of 528 individuals for *P. sitchensis* or 1,426 megagametophytes for *P. abies*), which resulted in improved marker ordering and genome anchoring (Bernhardsson et al. 2019; Tumas et al. 2024). Unfortunately, such sequencing/genotyping capacities remain limited for many research groups in the Global South, where most endemic, endangered conifers are distributed. We aim to increase the size of our mapping population in the near future, which may allow us to use our linkage map for genome assembly and functional genomics studies.

The reported figures from our *A. religiosa* linkage map translate into a genome-wide recombination rate of ∼0.086 cM/Mb, which is slightly lower than the average values reported for other conifers (typically ranging between 0.1 and 0.2 cM/Mbp; Jaramillo-Correa et al. 2010; Nystedt et al. 2013). This is consistent with the predicted relationship among genetic diversity, recombination, and effective population size (Charlesworth 2009), as most previously reported genome-wide recombination rates have been estimated for species with much broader distributions (and likely larger effective population sizes) than *A. religiosa* (ie *Picea glauca*, *Pinus taeda*, *Cryptomeria japonica*, etc.; Jaramillo-Correa et al. 2010).

### Functional co-localization of adjacent genes under ozone exposure

The concept of functional co-localization proposes that genes associated with the same metabolic pathway or phenotypic response are organized in close physical proximity (Boutanaev et al. 2002; Freeling 2009; Nazari and Zinati 2023). Such physical proximity facilitates their co-regulation and co-expression during adverse conditions, promoting a rapid response to threatening events (Nützmann and Osbourn 2014). This phenomenon has primarily been documented in model and cultivated plants, where clusters of genes linked to pathogen defense and oxidative stress have been observed (Ning et al. 2008; Short et al. 2012; Abedini and Rashidi Monfared 2018). Here, we showed that neighboring genes also tend to be co-expressed in several regions of the sacred fir linkage map, supporting the hypothesis that this co-regulation strategy also exists in conifers and may contribute to organizing efficient responses to ozone exposure. The regions involved are therefore of particular interest for conservation and management programs, as they could facilitate marker-based selection of tolerant genotypes for the reforestation of zones heavily affected by ozone pollution near Mexico City.

There was a clear connection between the differential expression observed between symptomatic and asymptomatic trees and the co-expression patterns observed within LGs, as several of the genes identified by edgeR/DESeq2 were located within regions with significant co-expression (ie LGs 5, 8, 10, and 11) and/or belonged to the same WGCNA module. The differences observed between symptomatic and asymptomatic trees were both in terms of magnitude and direction of the correlations (Fig. 4). Symptomatic trees showed co-expression clusters that included *carboxyesterase* genes on LG5 and *adenosylhomocysteinase*-like genes on LG10, which were less pronounced or absent in the asymptomatic plants. This scenario indicates that there are underlying differences in how certain metabolic pathways are coordinated between the 2 phenotypes.

The antagonistic expression observed in symptomatic trees, which was absent in asymptomatic plants (red arrows in Fig. 4), further indicates stronger transcriptional tradeoffs in symptomatic individuals. This suggests a more pronounced reorganization of transcriptional modules in symptomatic trees, in contrast to the more stable and less reactive networks of asymptomatic individuals. This leads to the hypothesis that asymptomatic individuals maintain their transcriptional state “primed” (eg Swindell 2006; Tarun et al. 2020; Subramani et al. 2023), while the symptomatic trees tend to transcriptionally “overreact,” potentially incurring higher metabolic costs and a less effective stress response. Detailed studies under controlled conditions are thus necessary to test this hypothesis and explore the (epi)genomic mechanisms underlying these transcriptional differences between asymptomatic and symptomatic firs.

### Modules of co-expressed genes putatively involved in ozone-stress response

Our results point to several regions within the sacred fir genome that warrant further resequencing efforts to better explore the genomic architecture underlying ozone-stress response. These regions (within LGs 5, 8, and 10) contain co-expressed neighboring genes whose function and annotation have been previously highlighted in studies with model plants. For instance, in LG5, 2 *carboxylesterase* genes (AB_013360_T.1 and AB_023350_T.1), separated by 3.7 cM, displayed similarly high expression during the high ozone period. In plants, carboxylesterases play a crucial detoxifying role by mediating the activation of the antioxidant system (Guidi et al. 2016; Rui et al. 2022); the overexpression of genes from this family has previously been reported in controlled ozone fumigation experiments in holm oaks (Natali et al. 2018).

In LG8, a pair of collocated genes, a *copper amine oxidase* and a gene coding for an unknown protein (AB_002192_T.1 and AB_007527_T.1), showed antagonistic expression in symptomatic plants when ozone levels increased. Copper amine oxidases are directly involved in peroxisomal metabolism and have been reported to be downregulated under ozone exposure in lentils and other plants (Maccarrone et al. 1997; Cona et al. 2006). Further investigation of the identity of this unknown gene and functionally testing its potential regulatory role would therefore be valuable.

Finally, in LG10, 2 *adenosylhomocysteinase* genes (AB_005854_T.1 and AB_024743_T.1), located only 0.6 cM apart, showed almost identical co-expression during the high ozone period. Adenosylhomocysteinases are involved in methylation processes and in adenosine metabolism; they form part of the stress response pathways, including ozone response, in *Arabidopsis* (Vannini et al. 2006), suggesting that they may modulate similar responses in conifers.

While the 3 genome regions identified in this study can be readily explored, they likely represent only a fraction of the genome zones involved in the response to ozone exposure in conifers (Reyes-Galindo et al. 2024). The linkage map presented here provides a string foundation for future QTL and genome-wide association studies using additional germplasm or controlled crosses, enabling the identification and mapping of loci involved in this complex response and their subsequent anchoring to the LGs and annotated regions reported here. Importantly, this work also represents one of the first attempts in conifers to integrate a dense linkage map with transcriptomic data (see also Pavy et al. 2017), thereby identifying regulatory hotspots and functionally co-expressed genes under environmental stress.

Although cross-species genome comparisons are still limited in tropical conifers—and in conifers in general—comparative mapping with species co-distributed with sacred fir near Mexico City (ie *Hesperocyparis benthamii*, *Pinus hartwegii,* and *P. patula*) could help determine whether similar genomic regions are involved in ozone response across species and allow testing hypotheses about the evolution of gene expression in conifers (eg Ghanbarian and Hurst 2015). In this sense, the candidate genes and gene networks putatively involved in ozone response reported here and in a previous study (from Reyes-Galindo et al. 2024) provide promising targets for validation in future functional and expression studies in *A. religiosa* and other conifers. This integrative strategy will promote the application of evolutionary genomics to the restoration of forests impacted by pollution, particularly for the conservation of threatened and understudied tropical montane conifers. For instance, we plan to develop a genotyping array based on the SNPs included in our linkage map, which could be used in future QTL analyses of ozone tolerance/sensitivity, as well as for preselecting tolerant germplasm in forest tree nurseries.

## Supplementary Material

jkag069_Supplementary_Data

## Data Availability

Code and scripts are archived on Zenodo (DOI: 10.5281/zenodo.17923287) and available on Xochitl Granados-Aguilar's GitHub at https://github.com/Xochitl-Citlalmina/Abies-linkage-map-transcriptome. Raw sequences for linkage map construction are available in GenBank (BioProject: PRJNA1314304). Supplementary material is available at G3 Online and includes Supplementary Figs. 1–5 and Supplementary Tables 1–5. Supplementary Fig. 1 shows dot plots for the 12 LGs of the composite map of *Abies religiosa;* Supplementary Fig. 2 the marker distributions across LGs compared with negative binomial and Poisson expectations; Supplementary Fig. 3 presents DESeq2-based gene expression profiles under contrasting ozone conditions; Supplementary Fig. 4 shows Pearson correlations between neighboring gene pairs across LGs, and Supplementary Fig. 5 shows WGCNA-based networks of co-localized and co-expressed genes. Supplementary Table 1 describes the *A. religiosa* samples used for RNA-seq analysis; Supplementary Table 2 lists annotated genes by-linkage group; Supplementary Table 3 provides functional annotations for 1,952 coding genes; Supplementary Table 4 contains sets of strongly correlated genes within LGs; and Supplementary Table 5 reports the *χ*² test results for the module-by-linkage group contingency table. Supplemental material available at G3 online.
