# Supplementary material for: Integrative linkage mapping and transcriptomic profiling uncover ozone-response modules in a peri-urban forest tree
Source: G3 (Bethesda). 2026 Mar 25;16(6):jkag069. doi: 10.1093/g3journal/jkag069 (PMC13261527; doi:10.1093/g3journal/jkag069)
Supplement: jkag069_Supplementary_Data [file jkag069_Supplementary_Data.zip › Supplementary_Legends_G3-2026-406677.docx]

**Supplementary Table and Figure Legends**

**Supplementary Table 1.** Description of A. religiosa samples used for gene expression analysis (RNA-seq; see Reyes-Galindo et al. 2024). The table includes the treatment ID, the tree from which it was obtained, the ozone level recorded during the sampling (high or moderate), and the presence/absence of foliar symptoms related to ozone stress (condition).

**Supplementary Table 2.** Genes annotated per linkage group according to locus number from STACKS and A. balsamea gene ID.

**Supplementary Table 3.** Detailed information on the 1,952 coding genes to which known protein functions were assigned using InterPro and TAIR databases. The table includes functional annotations for each gene, as summarized in Table 1.

**Supplementary Table 4.** Sets of strongly correlated genes (59-85 per linkage group) identified by analyzing expression value correlations among all gene pairs within the same linkage group.

**Supplementary Table 5.** Results of the chi-square test on the module-by-linkage group contingency table, showing the distribution of modules across the linkage map.

**Supplementary Figure 1.** Dot plots for each one of the 12 linkage groups of the composite map of Abies religiosa. Single edges indicate individual recombination; ellipses indicate multiple recombination events.

**Supplementary Figure 2.** Marker distribution on each one of the 12 linkage groups of the composite map of Abies religiosa along bins of (a) 10 cM and (b) 20 cM compared to a negative binomial distribution (red) and a Poisson distribution (blue).

**Supplementary Figure 3.** Gene expression profiles of symptomatic (damaged) and asymptomatic (healthy) Abies religiosa natural trees during two ozone (O₃) concentration periods, as determined with DESeq2 (see Fig. 3 for results obtained with edgeR). (a) Differential gene expression between symptomatic and asymptomatic trees during the high ozone period. (b) Comparison of gene expression between symptomatic and asymptomatic trees during the low ozone period. (c) Overall comparison of gene expression between symptomatic and asymptomatic trees, regardless of ozone concentration. (d) Comparison of gene expression profiles during the high and low ozone concentration periods, irrespective of the tree phenotype. Color scales represent normalized expression levels, with clustering indicating patterns of similarity across samples and genes.

**Supplementary Figure 4.** Pearson correlation coefficients (r) between the expression profiles of neighboring gene pairs (1 cM bins) along all linkage groups from the composite map of Abies religiosa. Correlations for the symptomatic trees are shown in blue, and correlations for the asymptomatic individuals are depicted in orange. Dashed lines represent critical significance values at α = 0.05. Red arrows indicate regions with strong negative correlations.

**Supplementary Figure 5.** Networks of co-localized and co-expressed in the linkage map of Abies religiosa based on WGCNA analysis.
