# Supplementary material for: Integrative linkage mapping and transcriptomic profiling uncover ozone-response modules in a peri-urban forest tree
Source: G3 (Bethesda). 2026 Mar 25;16(6):jkag069. doi: 10.1093/g3journal/jkag069 (PMC13261527; doi:10.1093/g3journal/jkag069)
Supplement: jkag069_Supplementary_Data [file jkag069_Supplementary_Data.zip › Supplementary_Table_1_G3-2026-406677.docx]

Supplementary Table 1. Description of *A. religiosa* samples used for gene expression analysis (RNA-seq; see Reyes-Galindo et al. 2024). The table includes the treatment ID, the tree from which it was obtained, the ozone level recorded during the sampling (high or moderate), and the presence/absence of foliar symptoms related to ozone stress (condition).

| Sample | ID | Level of O_3_ during sampling | Condition |
| --- | --- | --- | --- |
| Tree 1 symptomatic | DC01_15 | High | symptomatic |
| Tree 2 symptomatic | DC02_15 | High | symptomatic |
| Tree 3 symptomatic | DC03_15 | High | symptomatic |
| Tree 4 symptomatic | DC04_15 | High | symptomatic |
| Tree 5 symptomatic | DC05_15 | High | symptomatic |
| Tree 1 asymptomatic | SC01_15 | High | asymptomatic |
| Tree 2 asymptomatic | SC02_15 | High | asymptomatic |
| Tree 3 asymptomatic | SC03_15 | High | asymptomatic |
| Tree 4 asymptomatic | SC04_15 | High | asymptomatic |
| Tree 5 asymptomatic | SC05_15 | High | asymptomatic |
| Tree 1 symptomatic | DS01_15 | Moderate | symptomatic |
| Tree 2 symptomatic | DS02_15 | Moderate | symptomatic |
| Tree 4 symptomatic | DS04_15 | Moderate | symptomatic |
| Tree 1 asymptomatic | SS01_15 | Moderate | asymptomatic |
| Tree 2 asymptomatic | SS02_15 | Moderate | asymptomatic |
| Tree 5 asymptomatic | SS05_15 | Moderate | asymptomatic |
