# Supplementary figures and images for: Integrative linkage mapping and transcriptomic profiling uncover ozone-response modules in a peri-urban forest tree
Source: G3 (Bethesda). 2026 Mar 25;16(6):jkag069. doi: 10.1093/g3journal/jkag069 (PMC13261527; doi:10.1093/g3journal/jkag069)

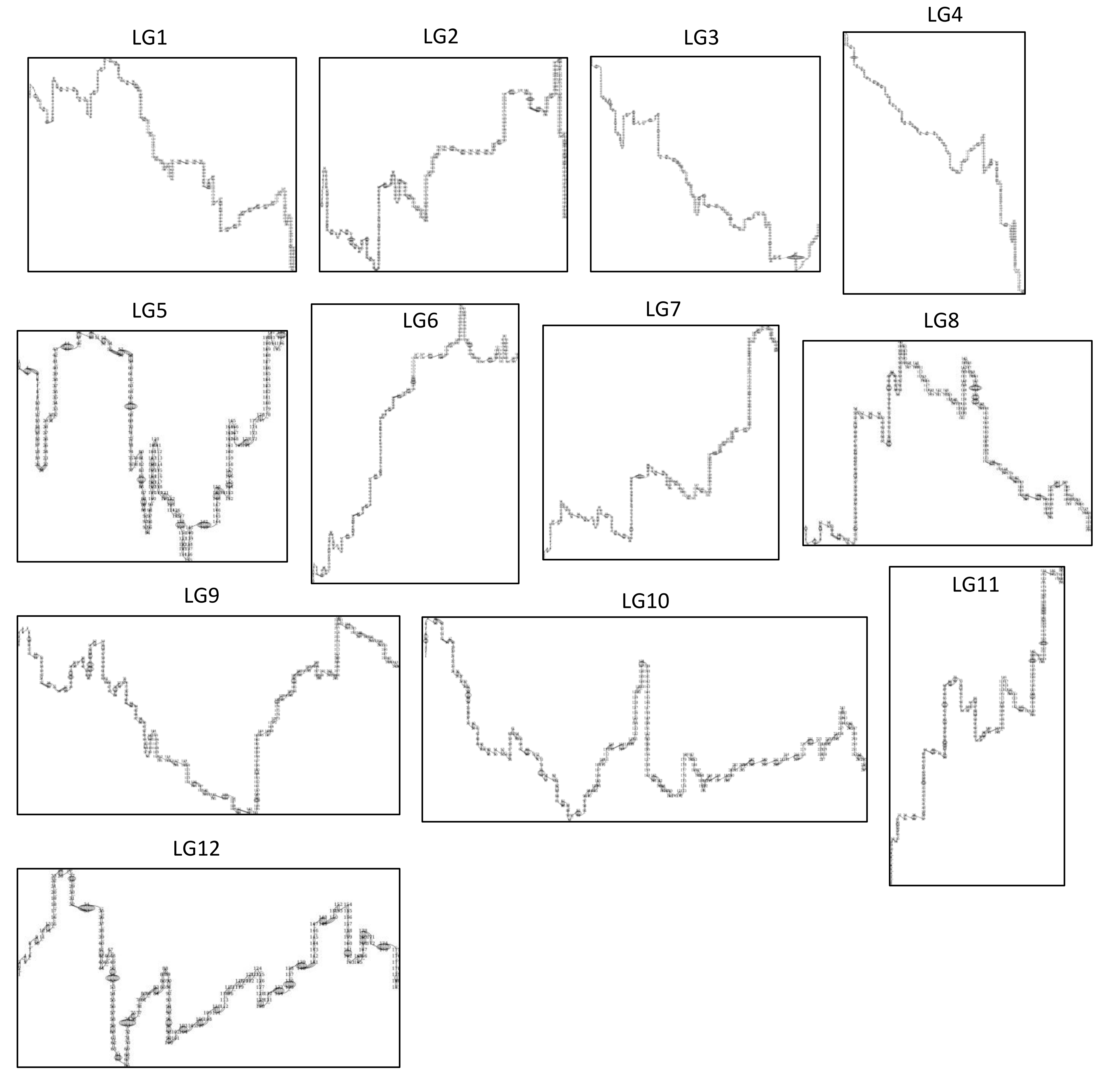

Supplement: jkag069_Supplementary_Data [file jkag069_Supplementary_Data.zip › Supplementary_Figure_1_G3-2026-406677.tif]

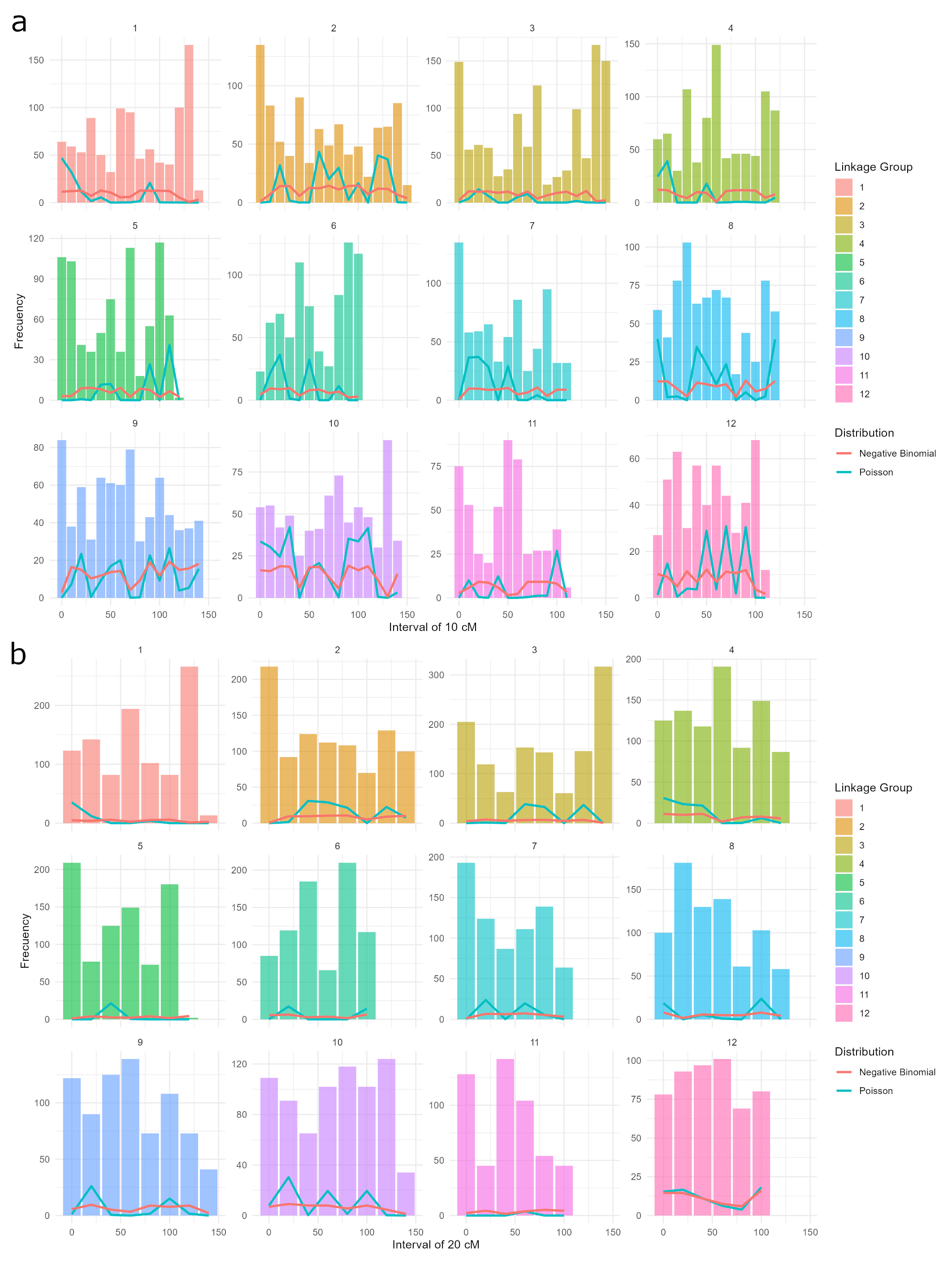

Supplement: jkag069_Supplementary_Data [file jkag069_Supplementary_Data.zip › Supplementary_Figure_2_G3-2026-406677.tif]

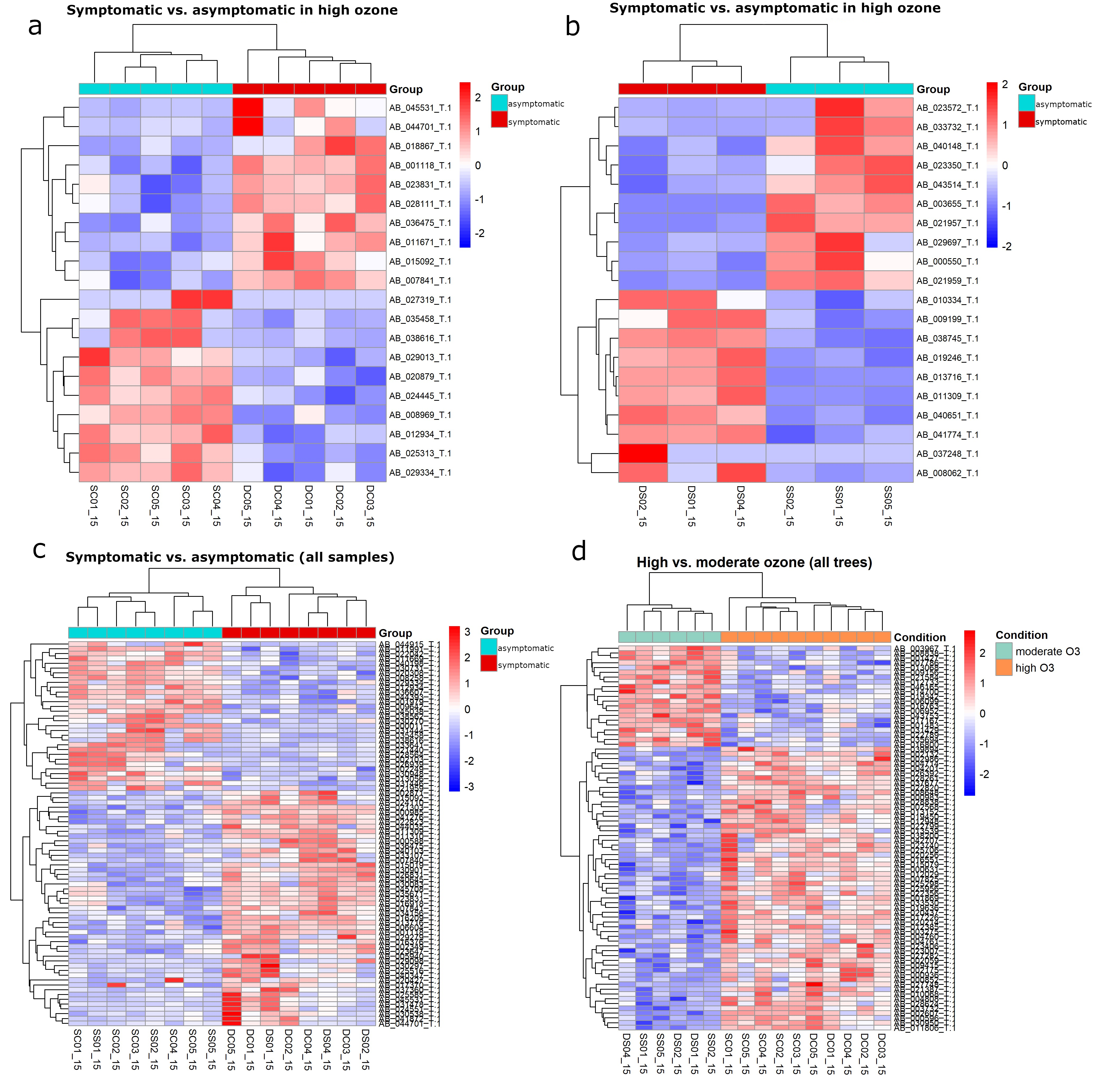

Supplement: jkag069_Supplementary_Data [file jkag069_Supplementary_Data.zip › Supplementary_Figure_3_G3-2026-406677.tif]

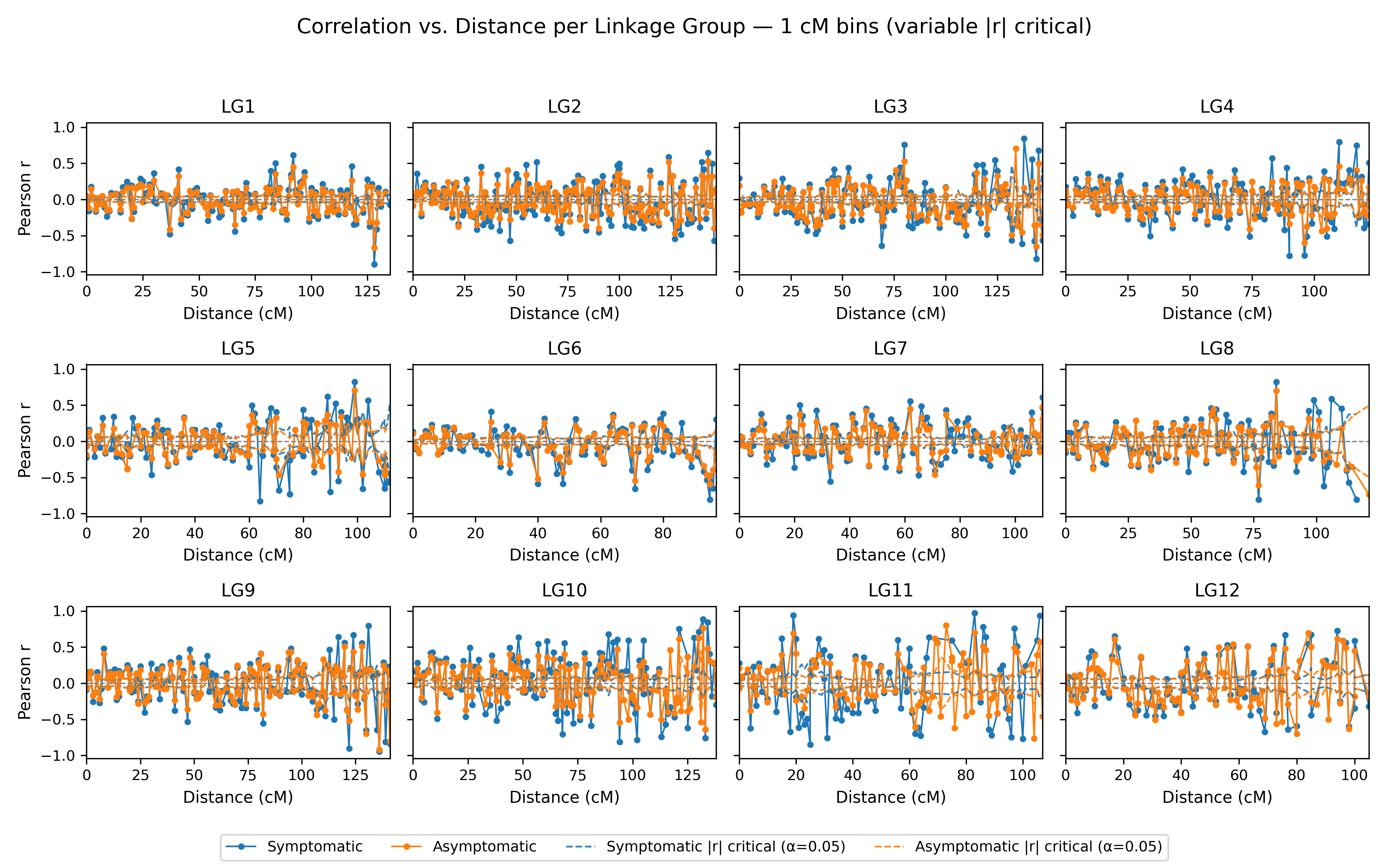

Supplement: jkag069_Supplementary_Data [file jkag069_Supplementary_Data.zip › Supplementary_Figure_4_G3-2026-406677.tif]

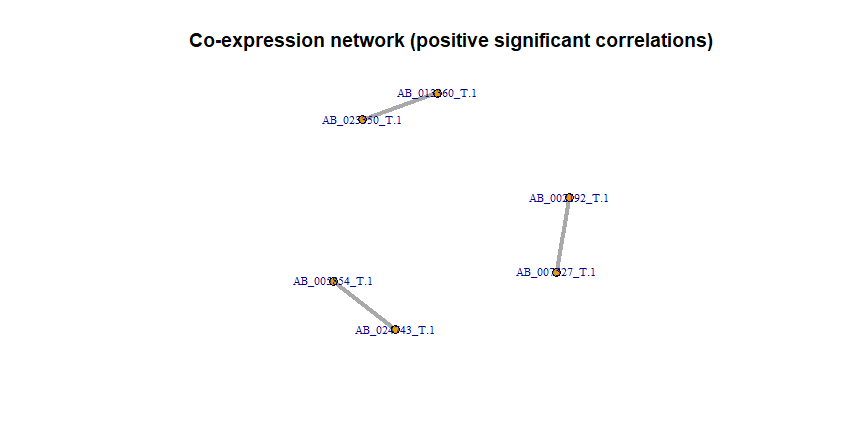

Supplement: jkag069_Supplementary_Data [file jkag069_Supplementary_Data.zip › Supplementary_Figure_5_G3-2026-406677.tif]
